# Diagnosis moderates the relationship between anxiety and digital communications in bipolar disorder and borderline personality disorder: A naturalistic remote-monitoring study

**DOI:** 10.1192/j.eurpsy.2021.922

**Published:** 2021-08-13

**Authors:** G. Gillett, N. Mcgowan, N. Palmius, A. Bilderbeck, G. Goodwin, K. Saunders

**Affiliations:** Department Of Psychiatry, University of Oxford, Oxford, United Kingdom

**Keywords:** Digital phenotyping, bipolar disorder, Borderline personality disorder, Anxiety

## Abstract

**Introduction:**

Differences in the relationship between mood and digital communication metrics have been shown to act as a diagnostic marker in Bipolar Disorder (BD) and Borderline Personality Disorder (BPD). Anxiety has been associated with mobile-phone use in non-clinical populations, although a potential association between anxiety and digital communications in BD or BPD populations hasn’t been studied.

**Objectives:**

To explore the association between self-reported anxiety symptoms and objective, naturalistic digital communications metrics in BD and BPD participants.

**Methods:**

BD (n= 17) and BPD (n=17) cohorts were provided with a smartphone application which monitored phone call and SMS frequency and duration, alongside weekly self-reported anxiety (Generalised Anxiety Disorder 7-item scale). Linear mixed-effects regression models assessed the association between digital communications, anxiety state and interaction effects between anxiety and diagnosis.

**Results:**

Self-reported anxiety state was negatively associated with decreased total call frequency (B=-5.150, p=0.002), cumulative total call duration (seconds; B=-1456.779, p<0.001), cumulative outgoing call duration (seconds; B=-1108.23, p<0.001), total SMS frequency (B=-31.412, p<0.001), outgoing SMS frequency (B=-16.443, p<0.001), cumulative total SMS length (characters; B=-1664.78, p=0.001) and cumulative outgoing SMS length (characters; B=-857.770, p=0.005) for BD, but not BPD, participants. Associations remained significant after adjusting for mood.

**Conclusions:**

These results further suggest that BPD individuals, compared to BD individuals, exhibit persistent social interaction during mental distress. Together with previous findings, this effect appears to be common, but independent, for both self-reported anxiety and depression. These findings inform our understanding of the psychopathology of the two conditions, and may contribute to the development of tools to aid their diagnostic differentiation.

**Conflict of interest:**

Prof Goodwin is a NIHR Emeritus Senior Investigator, holds shares in P1vital and P1Vital products and has served as consultant, advisor or CME speaker in the last 3 years for Compass pathways, Evapharm, Janssen, Lundbeck, Medscape, P1Vital, Sage, Servier.

